# The Immunomodulator 1-Methyltryptophan Drives Tryptophan Catabolism Toward the Kynurenic Acid Branch

**DOI:** 10.3389/fimmu.2020.00313

**Published:** 2020-02-28

**Authors:** Elisa Wirthgen, Anne K. Leonard, Christian Scharf, Grazyna Domanska

**Affiliations:** ^1^Department of Pediatrics, Rostock University Medical Center, Rostock, Germany; ^2^Institute of Biochemistry, University Medicine Greifswald, Greifswald, Germany; ^3^Department of Otorhinolaryngology, Head and Neck Surgery, University Medicine Greifswald, Greifswald, Germany; ^4^Institute of Immunology and Transfusion Medicine, University Medicine Greifswald, Greifswald, Germany

**Keywords:** 1-MT, IDO, KYNA, kynurenine pathway, tryptophan

## Abstract

**Background:** Animal model studies revealed that the application of 1-methyltryptophan (1-MT), a tryptophan (TRP) analog, surprisingly increased plasma levels of the TRP metabolite, kynurenic acid (KYNA). Under inflammatory conditions, KYNA has been shown to mediate various immunomodulatory effects. Therefore, the present study aims to confirm and clarify the effects of 1-MT on TRP metabolism in mice as well as in humans.

**Methods:** Splenocytes from Balb/C or indoleamine 2,3-dioxygenase knockout (*IDO1*^−/−^) mice or whole human blood were stimulated with 1-MT for 6, 24, or 36 h. C57BL/6 mice received 1-MT in drinking water for 5 days. Cell-free supernatants and plasma were analyzed for TRP and its metabolites by tandem mass spectrometry (MS/MS).

**Results:** 1-MT treatment induced an increase in TRP and its metabolite, KYNA in Balb/C, *IDO*^−/−^ mice, and in human blood. Concurrently, the intermediate metabolite kynurenine (KYN), as well as the KYN/TRP ratio, were reduced after 1-MT treatment. The effects of 1-MT on TRP metabolites were similar after the *in vivo* application of 1-MT to C57BL/6 mice.

**Conclusions:** The data indicate that 1-MT induced an increase of KYNA *ex vivo* and *in vivo* confirming previously described results. Furthermore, the results of *IDO*^−/−^ mice indicate that this effect seems not to be mediated by IDO1. Due to the proven immunomodulatory properties of KYNA, a shift toward this branch of the kynurenine pathway (KP) may be one potential mode of action by 1-MT and should be considered for further applications.

## Introduction

**Figure 1:**
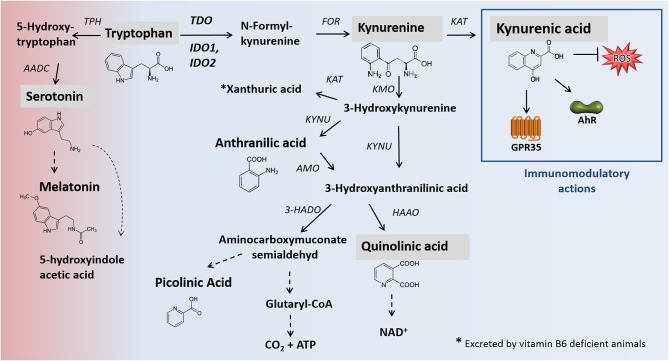
Main pathways of TRP degradation, including relevant enzymes [modified after (2, 3)]. Black arrows mark enzymatic reactions and dashed arrows include more than one catalytic reaction step. Metabolites analyzed in this study are shaded gray. The catabolism of TRP by the enzymes TDO, IDO1/IDO2 represents the rate-limiting step of the kynurenine pathway in which 95% of dietary TRP is oxidized. One percentage of dietary tryptophan for the synthesis of serotonin. Apart from neuromodulatory properties (not shown), KYNA is an agonist of the broadly expressed receptors GPR35 and AhR. Furthermore, KYNA functions as an ROS scavenger. KYNA production. In addition to the presented canonical pathway of KYNA formation, alternative routes of KYNA production, promoted by the presence of ROS, are described (4). AADC, aromatic L-amino acid decarboxylase; AANAT, N-acetyltransferase; AhR, hydrocarbon receptor; AMO, anthranilate 3-monooxygenase; FOR, formamidase; GPR35: G-protein-coupled receptor 35; HAAO, 3-hydroxyanthranilic acid oxidase; HADO, 3-hydroxyanthranilic acid 3,4-dioxygenase; IDO, indolamine 2,3-dioxygenase; KAT, kynurenine aminotransferase; KMO, kynurenine 3-monooxygenase; KYNU, kynureninase; TDO, tryptophan 2,3-dioxygenase; TPH, tryptophan hydroxylase; ROS, reactive oxygen species.

1-methyltryptophan (1-MT) is a TRP analog described first in 1991 (1) as a potential competitive inhibitor of the enzyme indoleamine 2,3-dioxygenase (IDO1). IDO1 is one rate-limiting enzyme of the kynurenine pathway (KP) (Figure 1), which plays a crucial role in the regulation of the immune response, notably as a counter-regulatory mechanism in the context of inflammation (2, 3). In cell free assays, it has been shown that 1-MT binds to the ferrous IDO complex but cannot be catalytically converted to kynurenine due to the additional methyl group (1). 1-MT is known for its low toxicity and great pharmacokinetic properties such as good intestinal absorption, low clearance, and low binding to plasmatic proteins (5, 6). Two stereoisomers of 1-MT, 1-methyl-D-tryptophan (D-1-MT also known as Indoximod) and 1-methyl-L-tryptophan (L-1-MT) are well studied and potential inhibitors of IDO. Indoximod is under investigation in several clinical trials (7–9) while L-1-MT or the combination of both isoforms DL-1-MT is used as IDO inhibitors in preclinical studies *in vivo* and *in vitro* (10–12). According to the reported IC_50_ values of L-1-MT (120 μM) and D-1-MT (2.5 mM) in HeLa cells (13), it is currently assumed that both L- and D-1-MT are weak IDO inhibitors *in vivo* (14, 15). Indeed, the D-isomer completely fails to inhibit the enzyme activity in inflammatory stimulated HeLa cells (9). The lack of IDO inhibition might be due to a low affinity of the inhibitor to the enzyme concurrent with a physiologically limited accumulation of the inhibitor to serum levels similar to those of TRP (16). Nevertheless, significant effects of these drugs on immune response were reported *in vivo* and *in vitro* (2, 16) revealing modes of actions other than IDO1 inhibition. Unexpectedly, the oral or subcutaneous applications of 1-MT in Balb/C mice (10) and pigs (11), resulted in increased plasma levels of the TRP metabolite kynurenic acid (KYNA), a stable end product of KP, rather than KYN, which is an intermediate metabolite of KP. Due to the proven immunomodulatory properties of KYNA (3), a shift of KP toward the KYNA branch may be one potential mode of action by 1-MT, which may also be relevant for the application in humans. As previously described in detail (3), under inflammatory conditions, KYNA mediates mainly immunosuppressive effects, notably by targeting the G-protein-coupled receptor 35 (GPR35)- or aryl hydrocarbon receptor (AhR)-associated signaling pathways (2, 17, 18). For instance, KYNA reduces the expression and secretion of TNFα (10, 19–21) and diminishes the secretion of high-mobility group box 1 in monocytes (20, 22). Furthermore, there is evidence that KYNA induces downregulation of IL23/IL17 axis (23), which is assumed to have beneficial effects as an anti-inflammatory treatment in many immune-mediated diseases (24). The anti-inflammatory effects of KYNA, as frequently observed in *in vitro* models, are also confirmed *in vivo* in mice. It has been shown that, in a mouse model of LPS-induced septic shock, KYNA treatment attenuated LPS induced pro-inflammatory mediators such as TNF-α and nitric oxide (NO) and significantly rescued animals from LPS-induced death (22, 25). It has been reported that the application of 1-MT in pigs resulted in increased plasma levels of KYNA of around 5 μM (11) which is sufficient to activate AhR and GPR35 as KYNA has a great affinity to the latter even at low micromolar range. In addition, these observations are further supported *ex vivo* in murine immune cells. Treatment of murine splenocytes with 5 μM KYNA exerted a slight proliferative effect concurrent with increased secretion of IL-1β and IL-6 (26), suggesting that 1-MT mediates its biological and yet significant effects via AhR and/or GPR35.

Taking the observed effects of 1-MT on KYNA production into consideration, the present study aimed at verifying these effects in other mouse strains as well as in humans. Furthermore, additional insights on other TRP metabolites are provided. We treated animals and/or human blood with 1-MT for different times. To further investigate if KYNA production is related to the activity of the IDO enzyme, the effect of 1-MT in IDO knockout mice was examined. In addition to *ex vivo* experiments, 1-MT was applied *in vivo* in the C57BL/6 mice to verify the previously described effects of 1-MT in Balb/C mice (10). TRP metabolism in cell culture supernatants or plasma was characterized by the metabolites of TRP and its downstream metabolites kynurenine (KYN), quinolinic acid (QUIN), KYNA, and serotonin (5-HT). As a marker for increased IDO1 activity, the ratio of KYN to TRP was calculated.

## Materials and Methods

### Animals

Female Balb/C (*n* = 6), *IDO1*^−/−^, and C57BL/6 mice (*n* = 41) were maintained in the breeding facility at the University of Greifswald, Germany. *IDO1*^−/−^ mice with a Balb/C background (*n* = 6) were provided by M. Moser (Brussels, Belgium) with the permission of A. Mellor (Augusta, Georgia, U.S.A.). As described in detail (27), the *IDO1*^−/−^ mice were generated using a DNA construct that targets the murine IDO gene in embryonic stem cells. The defective gene expression was verified in tissues with a high level of constitutive IDO1 expression by PCR and Western Blot. All mice were kept in groups (6–9 mice/cage) under controlled conditions with 12/12 light/dark cycle and access to food and water *ad libitum*. All animal experiments were approved by the Animal Protection Committee of Mecklenburg-Vorpommern, Germany (AZ 7221.3-1.1-083/12).

### Experiment 1: Stimulation of Murine Splenocytes and Human Whole Blood With 1-MT

Mice were euthanized as previously described (10). Spleens were aseptically removed into sterile, cold RPMI 1640 medium (Biochrom KG Berlin, Germany), and cell suspensions were prepared within 1 h after harvesting the organ. For *ex vivo* stimulation of whole blood cell cultures, human blood from six individual healthy donors was obtained from the Institute of Immunology and Transfusion Medicine, Greifswald after a written consent for scientific use. 1-MT (L-isomer, purity 95%; Sigma-Aldrich, Deisenhofen, Germany) was dissolved in 1 N NaOH to a stock concentration of 1M, and further dilutions were made in RPMI medium to a final concentration of 600 μM. A NaOH solvent control was prepared by adding 1 N NaOH to the cell culture medium to the same volume as used for 1-MT. After incubation with 1-MT for 6, 24, or 36 h. TRP and its metabolites were analyzed in cell-free supernatants by tandem mass spectrometry (MS/MS).

### Experiment 2: *in vivo* 1-MT Administration

1-MT (DL-isomer, 2 mg/ml) was given to C57BL/6 mice (*n* = 24) in the drinking water for 5 days. A control group (*n* = 17) received drinking water without 1-MT. After treatment, all animals were euthanized, blood was drawn by retroorbital puncture as previously described (10) into EDTA-tubes, plasma was separated and stored at −80°C until analysis of TRP and its metabolites.

### Quantification of 1-MT and TRP Metabolites

The determination of 1-MT, TRP, KYN, KYNA, and QUIN in plasma or cell-free supernatants was performed as previously described in detail (28, 29) using an API2000 tandem mass spectrometer equipped with an electrospray ion source (ABSciex, Darmstadt, Germany). As an indicator of IDO1 activation, the ratio of KYN and TRP (KYN × 100/TRP) was calculated (30).

### Statistics

Statistical analyses of experiment 1 was performed using SAS software, version 9.4 (SAS Institute Inc., Cary, NC, USA). The continuous response variables (TRP, KYN, KYNA, QUIN, 5-HT) were analyzed by Analysis of Variance (ANOVA) comprising the fixed effects treatment (1-MT, Control), and sampling time (6, 24, 36 h) and their interaction (treatment × time). In order to compare the six sets of measurements, the data were analyzed by fitting and testing generalized linear models applying the GLIMMIX procedure. The repeated statement in the GLIMMIX procedure was used with respect to repeated measurements on the same subject. Tukey–Kramer procedure was used for pair-wise multiple comparisons of TRP metabolites within one species. For the presentation of the results, the least square means (LS-means) and their standard errors (SE) were calculated and tested for each fixed effect in the model using the Tukey–Kramer procedure for all pair-wise comparisons. Data of experiment 2 were analyzed using the SigmaPlot 14.0 software. Differences between the treatment and the control group were compared with Welch's *t*-test or Mann-Whitney U test according to unequal variances of the treatment and the control group or failure of the normality test, respectively. The effects and differences were considered significant at *p* < 0.05.

## Results

### 1-MT Modifies TRP Metabolism and Increases KYNA *ex vivo*

To investigate whether 1-MT induces the production of KYNA *ex vivo*, murine splenocytes and human blood were stimulated with 1-MT for either 6, 24, or 36 h according to the experimental design 1. Due to the weak inhibitory potential of L-1-MT (IC_50_ = 120 μM) (13), a relatively high concentration i.e., 600 μM L-1-MT was chosen to achieve effective inhibition of IDO. To further investigate if KYNA production is dependent on a functional IDO enzyme, the effect of 1-MT in IDO knockout mice was examined. TRP and its metabolites were measured in supernatants of cell culture.

**Figure 2:**
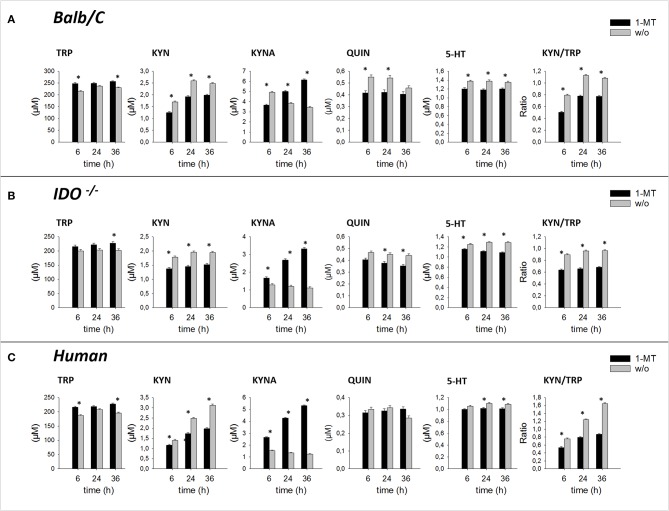
Effect of 1-MT on TRP metabolism in cultured splenocytes isolated from Balb/C mice **(A)**, *IDO*^−/−^ *mice* **(B)**, and in human whole blood cell culture **(C)**. TRP and its metabolites KYN, KYNA, QUIN were measured in cell culture supernatants after 6, 24, or 36 h incubation with 1-MT (600 μM) by MS/MS. KYN/TRP ratio was calculated as a marker for IDO1 activity. The results are presented as LS-means + SE. Significant differences between the 1-MT and the control group were calculated using the Tukey-Kramer test and are shown for each incubation time. *n* = 6 per species, *P < 0.05.

Pair-wise comparisons between 1-MT treatment and controls (main effect: Treatment) are presented in Table 1. Thereby, the concentrations of TRP were increased by 1-MT in Balb/C and *IDO1*^−/−^ mice as well as in human blood. That does not seem surprising since the applied L-1-MT contains 5% L-TRP, which corresponds to a concentration of 30 μM. In both murine and human cell culture supernatants, the increased level of TRP concentration corresponded to decreased levels of KYN, and 5-HT indicating downregulation of TRP breakdown. The downstream TRP metabolite QUIN was reduced by 1-MT in mice but not in human blood, suggesting species-specific differences. The observation of a diminished degradation of TRP via KP was further supported by the results of KYN to TRP ratio, often used as a marker for IDO activity (28). This ratio was decreased by 1-MT at all time points both in mice and human cell cultures, indicating that 1-MT inhibited the activity of IDO. Despite the presumed IDO inhibition by the high concentrations of 1-MT, the synthesis of kynurenine was not reduced completely, indicating a lack of effective IDO inhibition. The treatment effect on TRP and its metabolites KYN, 5-HT, and QUIN were comparable between different times of incubation with 1-MT (treatment × time, *p* > 0.05) (Figures 2A–C).

**Table 1:** Pairwise comparisons of the treatment effect (1-MT vs. control) using Tukey-Kramer test.

	**1-MT**	**Control**	**SE**	***P*-value**
	**LSM**	**LSM**		
**TRP (μM)**				
Balb/C	250.94	227.44	3.24	**0.003**
IDO1^−/−^	221.61	202.00	4.43	**0.030**
Human	220.78	197.00	5.08	**0.002**
**KYN (μM)**				
Balb/C	1.72	2.26	0.11	**0.001**
IDO1^−/−^	1.45	1.90	0.07	**0.001**
Human	1.62	2.34	0.18	**0.001**
**KYNA (μM)**				
Balb/C	4.95	4.06	1.05	**0.047**
IDO1^−/−^	2.56	1.20	0.16	** <0.001**
Human	4.09	1.37	0.29	** <0.001**
**QUIN (μM)**				
Balb/C	0.42	0.52	0.02	**0.002**
IDO1^−/−^	0.38	0.45	0.01	** <0.001**
Human	0.33	0.32	0.01	0.64
**5-HT (μM)**				
Balb/C	1.20	1.37	0.03	** <0.001**
IDO1^−/−^	1.12	1.28	0.02	** <0.001**
Human	1.01	1.08	0.01	**0.002**
**KYN/TRP ratio**				
Balb/C	0.69	1.00	0.06	**0.001**
IDO1^−/−^	0.66	0.94	0.03	** <0.001**
Human	0.73	1.22	0.12	**0.002**

Concentrations of the metabolites TRP, KYN, KYNA, QUIN, 5-HT were measured in cell culture supernatants of isolated splencocytes (Balb/C, IDO1^−/−^) and human whole blood cell culture. As marker for IDO activity KYN/TRP ratio was calculated. Results are presented as LS-means (LSM) ± SE averaged over three incubation times (6, 24, 36 h) per subject. 1-MT: n = 18 (six per species); Control: n = 18 (six per species).

P-values < 0.05 are marked in bold.

1-MT induced an increase of KYNA in cell culture supernatants of murine splenocytes as well as in human blood, confirming studies in pigs and mice (10, 11). Interestingly, this effect was also detected in *IDO1*^−/−^ mice, revealing that KYNA production was not related to a functional IDO enzyme. In splenocytes of *IDO1*^−/−^ mice and human blood, increased levels of KYNA were detected after 6, 24, and 36 h of stimulation with 1-MT, indicating a TRP breakdown toward the KYNA branch. Interestingly, in Balb/C mice, KYNA levels were lower in the treatment group compared to controls after 6 h of stimulation with 1-MT (treatment × time, *p* < 0.05). However, this effect might not reflect a reduced KYNA production but is rather the consequence of an increased KYNA concentration in the control group.

### 1-MT Modifies TRP Metabolism and Increases KYNA *in vivo*

**Figure 3:**
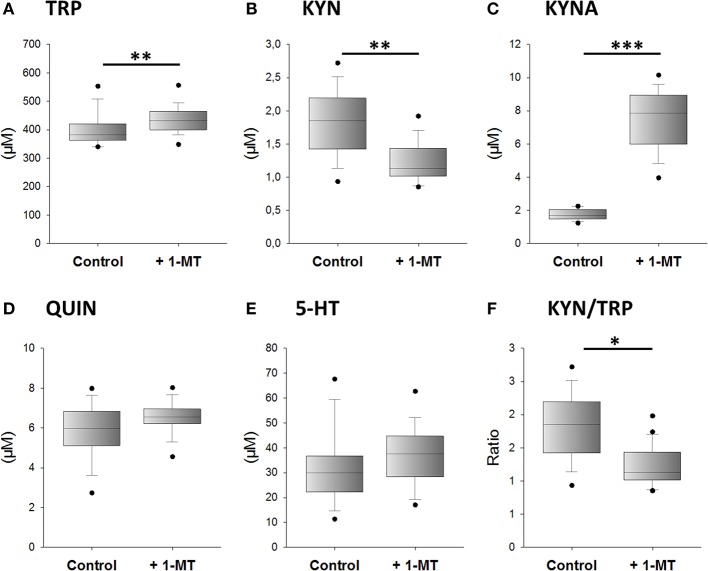
Effects of 1-MT application on plasma concentrations of TRP **(A)**, KYN **(B)**, KYNA **(C)**, QUIN **(D)**, 5-HT **(E)**, and the calculated KYN/TRP ratio **(F)**. 1-MT was applied via drinking water for 5 days. Control animals received drinking water without 1-MT. TRP and its metabolites were measured using MS/MS. The KYN/TRP ratio was used as a marker for IDO1 activity. The results are presented as boxplot including the median, the interquartile range, and the min and max values. Significant differences between the 1-MT- and control groups were calculated using the Welsh's test **(B–F)** or the Mann-Whitney U test **(A)**. 1-MT: *n* = 24; MYR: *n* = 17; ***P < 0.001, **P < 0.01, *P < 0.05.

We next investigated whether 1-MT induces the production of KYNA after *in vivo* application. According to the design of experiment 2, 1-MT was given to C57BL/6 in drinking water over a period of 5 days. Afterward, TRP and its metabolites were measured in plasma. The results are presented in Figure 3. After 5 days, 1-MT concentration in plasma was reached to ~25.5 μM, whereas in control animals the concentration was below the detection range (LLOQ: <2.3 μM) (data are not shown). The ratio of KYN to TRP was decreased after 1-MT treatment indicating a diminished TRP breakdown. However, it is assumed that the plasma concentrations achieved *in vivo* do not effectively block IDO activity, indicating that this effect is not related to the activity of the IDO enzyme. Similar to *ex vivo* findings, the pairwise comparisons reveal that the 1-MT treatment increased plasma levels of TRP and KYNA. In contrast, the concentrations of QUIN and 5-HT were not significantly affected by 1-MT treatment.

Together with the previously published findings from mice and pigs, the present study reveals that 1-MT induces an increase of KYNA concentrations in different species, including humans. Furthermore, these effects seem to be independent of IDO activity.

## Discussion

In this study, effects of 1-MT on TRP metabolism were investigated in mice and humans to provide additional insights into potential modes of action of 1-MT as previously described in mice and pigs (10, 11). The results of experiment 1 show that 1-MT increased the concentrations of TRP in supernatants of cultured murine splenocytes, human blood cell culture, and murine plasma. These results confirm the data from pigs where plasma concentrations of TRP are increased after 24 h of treatment with 1-MT subcutaneously (11). The effect of increased TRP concentrations may be a result of indirect supplementation of TRP contained in commercially available 1-MT, though the applied 1-MT has a purity of at least 95%. As confirmed by previous studies, 1-MT contains ≤ 5% TRP (11, 31), which may affect the results of *in vitro* or *ex vivo* studies, independently of IDO activity. However, the indirect TRP supplementation does not sufficiently explain the results *in vivo* because the amount of TRP, contained in 1-MT is negligible compared to the dietary derived TRP amounts, which are positively regulated by tryptophan 2,3-dioxygenase (TDO) (mainly expressed in the liver) to maintain the homeostasis of TRP (32). It has been further described that both isomers of 1-MT mimic TRP (14, 33), which may feign an amino acid oversupply and, therefore, may reduce the uptake of endogenous TRP to somatic cells resulting in increased TRP levels. Although it could not be clarified conclusively whether TRP contamination of 1-MT and/or other effects such as TRP mimetics caused the increase of TRP, the summary of results from previous studies, including the present study, indicate that it seems to be a general effect. Since higher TRP concentrations lead to an activation of TDO, one can assume that TRP supplementation may result in an accelerated TRP degradation concurrent with increased production of TRP downstream metabolites. For instance, in human glioblastoma cells, the TRP contamination of 1-MT (L-isomer) resulted in an increased production of KYN, indicating an accelerated degradation of TRP via KP (31). This was supported by studies in pigs where TRP supplementation resulted in an overall activation of the KP as well as the 5-HT system (34). In contrast, the results of our present study show that the 1-MT-induced elevation of TRP, which was associated with increased production of KYNA with a decrease of other TRP metabolites such as KYN or 5-HT in mice as well as in human blood. Also, the ratio of KYN to TRP, often used as a marker for IDO activity, was decreased in all investigated species after both *ex vivo* and *in vivo* application of 1-MT. In the present study, these results reveal an enhanced degradation of TRP directed to one specific branch of the KP (see also Figure 1) which not only seems to be a consequence of increased TRP levels but is also related to 1-MT. Although in the present study, the effect of 1-MT on KYN to TRP ratio seems to be uniform between *ex vivo* and *in vivo* studies, the *in vivo* treatment of healthy Balb/C mice (10) or pigs (11) did not significantly affect the KYN to TRP ratio indicating species/strain-specific responses to 1-MT or effects of the experimental design. Nevertheless, the results from mice and pigs (10, 11) showed independently from each other that 1-MT induced an increase of KYNA indicating that this effect is not restricted to a specific animal model. Furthermore, the findings in human blood indicate that this effect may be even relevant for the application of 1-MT in humans.

The finding that 1-MT induced an elevation of KYNA, but not the intermediate metabolite KYN, raises the question of whether TRP is metabolized directly to KYNA rather than through IDO activity. Computational modeling of data evaluated from a previously reported study in pigs (11) reveals that it is more likely that increased levels of TRP are directly degraded to KYNA (35). It is assumed that the major production of KYNA is attributed to kynurenine aminotransferases (KATs), enzymes with a broad spectrum of substrates besides KYN. *In vitro* analysis show that TRP is a substrate for the enzyme KAT II with a similar *Km*-value as the substrate KYN (36). Interestingly, in the present study, KYNA was also increased in IDO knockout mice, supporting the hypothesis that this effect is not related to a functional IDO enzyme. This is supported by the finding that increased KYNA levels were also measured after *in vivo* application of 1-MT, although the plasma concentrations of 1-MT (~25 μM) were too low to inhibit IDO effectively.

The finding that other TRP metabolites of KP or serotonin pathway are decreased, or unaffected indicates that the KYNA production is not mediated by one of the rate-limiting enzymes of KP (canonical pathway) but might be mediated by other TRP degrading enzymes or non-enzymatically. According to this hypothesis, alternative mechanisms of KYNA production (4) might be involved. For instance, it was described that enzymes such as the aromatic amino acid transaminase are able to catalyze TRP to intermediate metabolites which serve as KYNA precursors in the presence of reactive oxygen radicals (ROS) (4).

The theory that increased TRP may promote KYNA production was tested in an additional experiment. Thereby, the application of 5% TRP (contained in L-1-MT) induced an increase of KYNA production 3 h after application but not after longer incubation time, indicating that the additional TRP is metabolized via the kynurenine pathway (data not shown). However, the KYNA production by L- and D-1-MT was 2 to 3-fold higher compared to L-TRP alone, which leads one to assume that the TRP contamination is not an exclusive explanation for the increased KYNA values. According to this, it is suggested that other mechanisms might be involved in the increased production of KYNA. Although there is currently a lack of data, it cannot be ruled out that 1-MT itself is metabolized or transformed to TRP or other TRP related metabolites by a yet unknown pathway. These questions remain to be clarified in further studies.

In the present study, it seems surprising that the loss of functional IDO1 in the IDO KO mice has no effect on the 1-MT-induced changes of TRP metabolites. These data implicate that other tryptophan-catabolizing enzymes like IDO2 and tryptophan 2,3-dioxygenase (TDO) might compensate for the lack of IDO1. This theory is supported by other studies demonstrating that IDO1 deficiency does not affect the inflammation in murine models of arthritis, pregnancy, or bacterial infection (27, 37, 38).

## Conclusions

The results from the present study indicate that the administration of 1-MT induces a shift of KP toward the branch of KYNA. This effect was shown independently in two mice strains as well as in human blood. Furthermore, the results from IDO knockout mice, as well as the decreased levels of KYN *in vivo*, indicate that the 1-MT-induced increase of KYNA seems not to be dependent on IDO1 activity. This was confirmed by the *in vivo* results where 1-MT levels were too low for an effective IDO1 inhibition; nevertheless, KYNA was increased in plasma. The increase of KYNA may be one potential mode of action by 1-MT and should be considered for preclinical studies and therapeutic applications in humans. Furthermore, the application of 1-MT might have therapeutic implications as it may provide a method to increase KYNA while preserving IDO1 activity. Due to its immunoregulatory role, the inhibition of IDO1 may not be appropriate for different therapeutic approaches.

## Data Availability Statement

The datasets generated for this study are available on request to the corresponding author.

## Ethics Statement

Ethical review and approval was not required for the study on human participants in accordance with the local legislation and institutional requirements. The patients/participants provided their written informed consent to participate in this study. All animal experiments were approved by the Animal Protection Committee of Mecklenburg-Vorpommern, Germany (AZ 7221.3-1.1-083/12).

## Author Contributions

GD conceived the project, designed and performed the experiments, and analyzed MS data. GD and EW wrote the manuscript. EW analyzed the data, created the figures, and wrote the first draft of the manuscript. AL and CS assisted with performing of the MS analysis and critically revised the manuscript for important intellectual content. All authors critically reviewed the manuscript and approved the final version.

### Conflict of Interest

The authors declare that the research was conducted in the absence of any commercial or financial relationships that could be construed as a potential conflict of interest.
